# Supplementary material for: Change and stasis of distinct sediment microbiomes across Port Everglades Inlet (PEI) and the adjacent coral reefs
Source: PeerJ. 2023 Jan 13;11:e14288. doi: 10.7717/peerj.14288 (PMC9841897; doi:10.7717/peerj.14288)
Supplement: Supplemental Information 1 [file peerj-11-14288-s001.docx]

**Table S1** - Nutrients and chemicals analyzed by FIU indicated in last column of Table 1

| **Analyte** | **Type of sample processed** |
| --- | --- |
|  |  |
| Nitrate-nitrite | Filtered water sample |
| Ammonium | Filtered water sample |
| Soluble Reactive Phosphorus | Filtered water sample |
| Nitrite | Filtered water sample |
| Total Organic Carbon | Whole water sample |
| Dissolved Organic Carbon | Whole water sample |
| Trace Metals | Sediment sample |
| Soil Total Phosphorus | Sediment sample |
| Soil Total Carbon | Sediment sample |
| Total Nitrogen | Sediment sample |
