# Supplementary material for: Change and stasis of distinct sediment microbiomes across Port Everglades Inlet (PEI) and the adjacent coral reefs
Source: PeerJ. 2023 Jan 13;11:e14288. doi: 10.7717/peerj.14288 (PMC9841897; doi:10.7717/peerj.14288)
Supplement: Supplemental Information 2 [file peerj-11-14288-s002.docx]

***Table S2*** *– 2020 and 2021 sample IDs and Number of 16S rRNA sequences (reads) for each 2021 port and reef sample. The first three alphanumeric symbols of each ID match the sampling sites on the map of Figure 1.*

|  | 2020 | 2021 |
| --- | --- | --- |
| **Sample** | **# of MiSeq Reads** | **# of MiSeq Reads** |
| R01.00 | 110919 | 81155 |
| R01.15 | 76478 | 85722 |
| R01.30 | 88076 | 83627 |
| R02.00 | 153038 | 87274 |
| R02.15 | 123225 | 82831 |
| R02.30 | 138646 | No data |
| R03.00 | 104145 | No data |
| R03.15 | 88603 | 90168 |
| R03.30 | 84058 | 103571 |
| R04.00 | 68290 | 65912 |
| R04.15 | 129641 | 82689 |
| R04.30 | 150941 | 73018 |
| R05.00 | 128490 | 131002 |
| R05.15 | 156998 | 81090 |
| R05.30 | 130199 | 121775 |
| R06.00 | 118470 | No data |
| R06.15 | 122263 | 121350 |
| R06.30 | 110059 | 142681 |
| R07.00 | 146563 | 131947 |
| R07.15 | 145192 | 67200 |
| R07.30 | 141461 | No data |
| R08.00 | 122697 | 99057 |
| R08.15 | 125603 | 76815 |
| R08.30 | 139606 | 86341 |
| R09.00 | 106442 | 78290 |
| R09.15 | 129984 | 83030 |
| R09.30 | 97402 | 64428 |
| R10.00 | 97053 | 90466 |
| R10.15 | 100247 | 56536 |
| R10.30 | 108609 | 75904 |
| R11.00 | 97651 | 98635 |
| R11.15 | 71320 | 68434 |
| R11.30 | 79481 | 110112 |
| R12.00 | 105947 | 63745 |
| R12.15 | 56187 | 73422 |
| R12.30 | 24941 | 106510 |
| R13.00 | 154202 | 107331 |
| R13.15 | 153036 | 99834 |
| R13.30 | 142149 | 101661 |
| R14.00 | 141240 | 101166 |
| R14.15 | 113913 | 102714 |
| R14.30 | 129684 | 83771 |
| R15.00 | 65336 | 148804 |
| R15.15 | 52797 | 15587 |
| R15.30 | 74246 | 124965 |
| R16.00 | 68059 | 74928 |
| R16.15 | 133624 | 88591 |
| R16.30 | 118907 | 116891 |
| R17.00 | 131095 | 122739 |
| R17.15 | 137757 | 92756 |
| R17.30 | 96914 | 88733 |
| R18.00 | 136007 | 70488 |
| R18.15 | 119347 | 119212 |
| R18.30 | 115883 | 135836 |
| R19.00 | 146257 | 53585 |
| R19.15 | 145495 | 79812 |
| R19.30 | 128171 | 71580 |
| R20.00 | 149611 | 77116 |
| R20.15 | 134315 | 125653 |
| R20.30 | 113688 | 128382 |
| R21.00 | 140647 | 85906 |
| R21.15 | 141277 | 118126 |
| R21.30 | 118015 | 130385 |
| R22.00 | 141631 | 136255 |
| R22.15 | 119686 | 115038 |
| R22.30 | 146611 | 146674 |
| P01.0 | 62035 | 149017 |
| P01.15 | 84928 | 113930 |
| P01.30 | 87024 | 138808 |
| P02.00 | 66235 | 193324 |
| P02.15 | 123068 | 157524 |
| P02.30 | 125196 | 156797 |
| P03.0 | 131318 | 210425 |
| P03.15 | 114132 | 93428 |
| P03.30 | 73738 | 185206 |
| P04.00 | 139697 | 133747 |
| P04.15 | 101726 | 125556 |
| P04.30 | 139890 | 97354 |
| P05.0 | 120628 | 163624 |
| P05.15 | 87721 | 106239 |
| P05.30 | 125224 | 180446 |
| P06.00 | 134124 | 181295 |
| P06.15 | 144549 | 124471 |
| P06.30 | 119151 | 105951 |
| P07.0 | 78282 | 50594 |
| P07.15 | 129698 | 60561 |
| P07.30 | 333 | 94932 |
| P08.00 | 103928 | 119552 |
| P08.15 | 91529 | No data |
| P08.30 | 108782 | No data |
| P09.00 | 125077 | 143719 |
| P09.15 | 103048 | 203652 |
| P09.30 | 80154 | 165775 |
| P10.00 | 124786 | 197909 |
| P10.15 | 80122 | 174419 |
| P10.30 | 85819 | 145567 |
| P11.00 | 139222 | 142131 |
| P11.15 | 155034 | 208506 |
| P11.30 | 95545 | 192905 |
| P12.00 | 76183 | 229599 |
| P12.15 | 125736 | 204089 |
| P12.30 | 131358 | 194495 |
| P13.00 | 130592 | 192539 |
| P13.15 | 129974 | 155594 |
| P13.30 | 136056 | No data |
| P14.00 | 138561 | 229308 |
| P14.15 | 103582 | 219848 |
| P14.30 | 129000 | 213096 |
| P15.00 | 91808 | 217162 |
| P15.15 | 143938 | 149734 |
| P15.30 | 128424 | 144116 |
| P16.00 | 111556 | 183071 |
| P16.15 | 79326 | 155733 |
| P16.30 | 63928 | 189510 |
| P17.00 | 119129 | 166491 |
| P17.15 | 116944 | 156619 |
| P17.30 | 123328 | 205810 |
| P18.00 | 278512 | 222666 |
| P18.15 | 136324 | 179254 |
| P18.30 | 122814 | 51611 |
|  |  |  |
|  |  |  |

Highlighted samples indicated those omitted in final analyses. “.00, 0.15 and 0.30” indicate the distance of the site marker, and comprise the 3 replicates per site.

Three 2020 samples remained problematic, and could not be included in final analyses because reads appeared either too high (P18-Mi-00 with >240,000 reads) or too low (R12.30 had < 25,000 reads; P07.30 had <1000 reads). Seven samples from 2021 failed and thus not included in the final analyses.
