# Supplementary material for: Change and stasis of distinct sediment microbiomes across Port Everglades Inlet (PEI) and the adjacent coral reefs
Source: PeerJ. 2023 Jan 13;11:e14288. doi: 10.7717/peerj.14288 (PMC9841897; doi:10.7717/peerj.14288)
Supplement: Supplemental Information 3 — The first three alphanumeric symbols of each ID match the sampling sites on the map of Figure 1. [file peerj-11-14288-s003.docx]

**Table S3. P values for each comparison of trace metal concentrations**

|  | V | Cr | Mn | Fe | Co | Ni | Cu | Zn |
| --- | --- | --- | --- | --- | --- | --- | --- | --- |
|  |  |  |  |  |  |  |  |  |
| port 2020 vs. reef2020 | 0.6288 | 0.2399 | 0.1851 | 0.0124 | 0.0124 | 0.0003 | <0.0001 | <0.0001 |
| port 2020 vs. port2021 | 0.9862 | 0.9976 | 0.489 | 0.818 | 0.818 | 0.9729 | 0.8963 | 0.999 |
| port 2020 vs. reef2021 | 0.7755 | 0.3415 | 0.2157 | 0.0043 | 0.0043 | 0.0004 | <0.0001 | <0.0001 |
| reef2020 vs. port2021 | 0.408 | 0.1711 | 0.964 | 0.0806 | 0.0806 | 0.0008 | <0.0001 | <0.0001 |
| reef2020 vs. reef2021 | 0.9887 | 0.9902 | 0.9994 | 0.9026 | 0.9026 | 0.9989 | 0.9644 | 0.3925 |
| port2021 vs. reef2021 | 0.5505 | 0.2508 | 0.9813 | 0.0291 | 0.0291 | 0.001 | <0.0001 | <0.0001 |
|  |  |  |  |  |  |  |  |  |
|  | As | Se | Ba | Pb | Be | Al | Mo | Cd |
| port 2020 vs. reef2020 | 0.9998 | 0.0004 | 0.1046 | <0.0001 | 0.0002 | <0.0001 | 0.0334 | 0.8142 |
| port 2020 vs. port2021 | 0.9934 | 0.1057 | 0.5282 | 0.7042 | 0.996 | 0.7257 | 0.9263 | 0.4482 |
| port 2020 vs. reef2021 | 0.9441 | 0.032 | 0.0796 | <0.0001 | 0.0066 | <0.0001 | 0.0639 | 0.33 |
| reef2020 vs. port2021 | 0.9956 | <0.0001 | 0.808 | <0.0001 | 0.0002 | <0.0001 | 0.0088 | 0.8142 |
| reef2020 vs. reef2021 | 0.8674 | 0.044 | 0.9974 | 0.7901 | 0.0971 | 0.982 | 0.9717 | 0.7068 |
| port2021 vs. reef2021 | 0.8272 | 0.0002 | 0.7239 | <0.0001 | 0.0107 | <0.0001 | 0.0171 | >0.9999 |
|  |  |  |  |  |  |  |  |  |
