# Supplementary material for: Change and stasis of distinct sediment microbiomes across Port Everglades Inlet (PEI) and the adjacent coral reefs
Source: PeerJ. 2023 Jan 13;11:e14288. doi: 10.7717/peerj.14288 (PMC9841897; doi:10.7717/peerj.14288)
Supplement: Supplemental Information 4 — Symbol shapes and color match Figure 3. Clustering is denoted by the green solid lines at 80%. [file peerj-11-14288-s004.pdf]

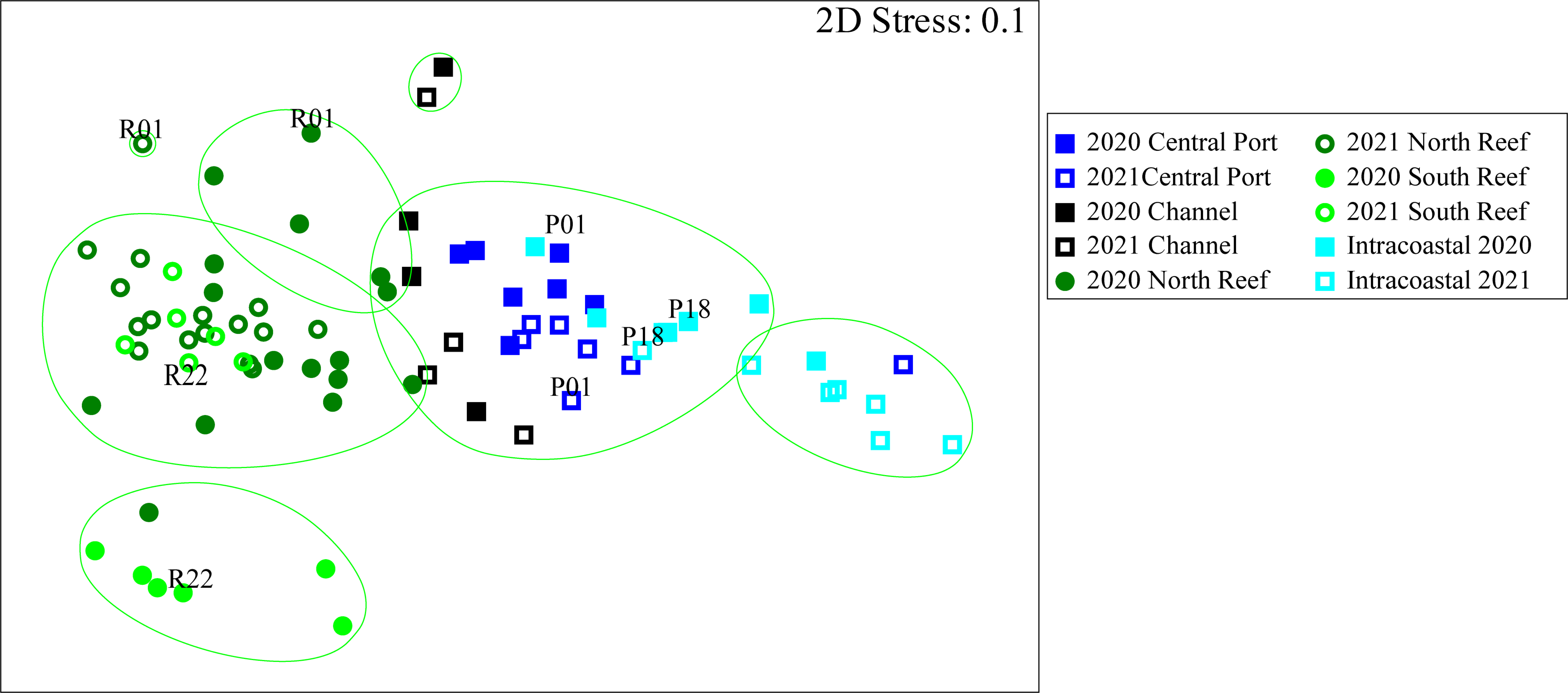
2D Stress: 0.1
R01
R01
P01
P18
P18
P01
R22
R22
2020 Central Port
2021Central Port
2020 Channel
2021 Channel
2020 North Reef
2021 North Reef
2020 South Reef
2021 South Reef
Intracoastal 2020
Intracoastal 2021
