# Supplementary figures and images for: Change and stasis of distinct sediment microbiomes across Port Everglades Inlet (PEI) and the adjacent coral reefs
Source: PeerJ. 2023 Jan 13;11:e14288. doi: 10.7717/peerj.14288 (PMC9841897; doi:10.7717/peerj.14288)

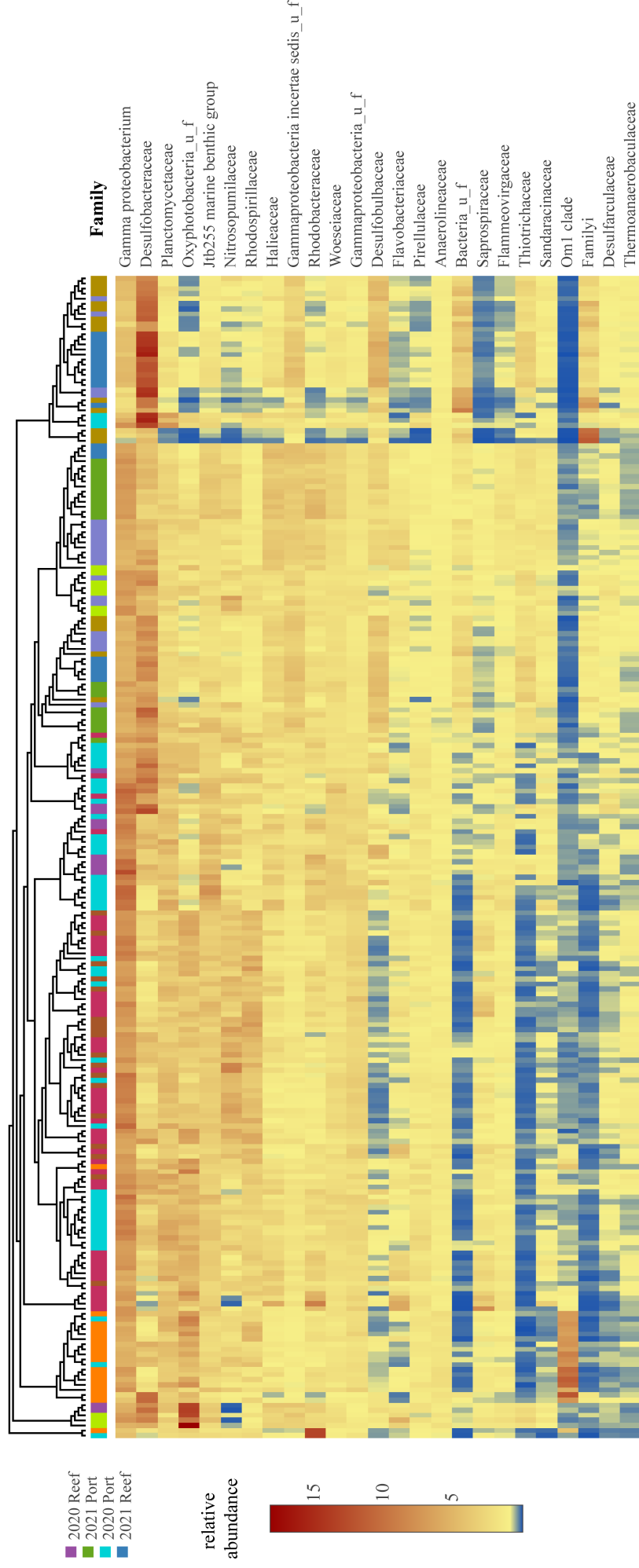

Supplement: Supplemental Information 5 — Samples are clustered based on abundance profiles. [file peerj-11-14288-s005.pdf]

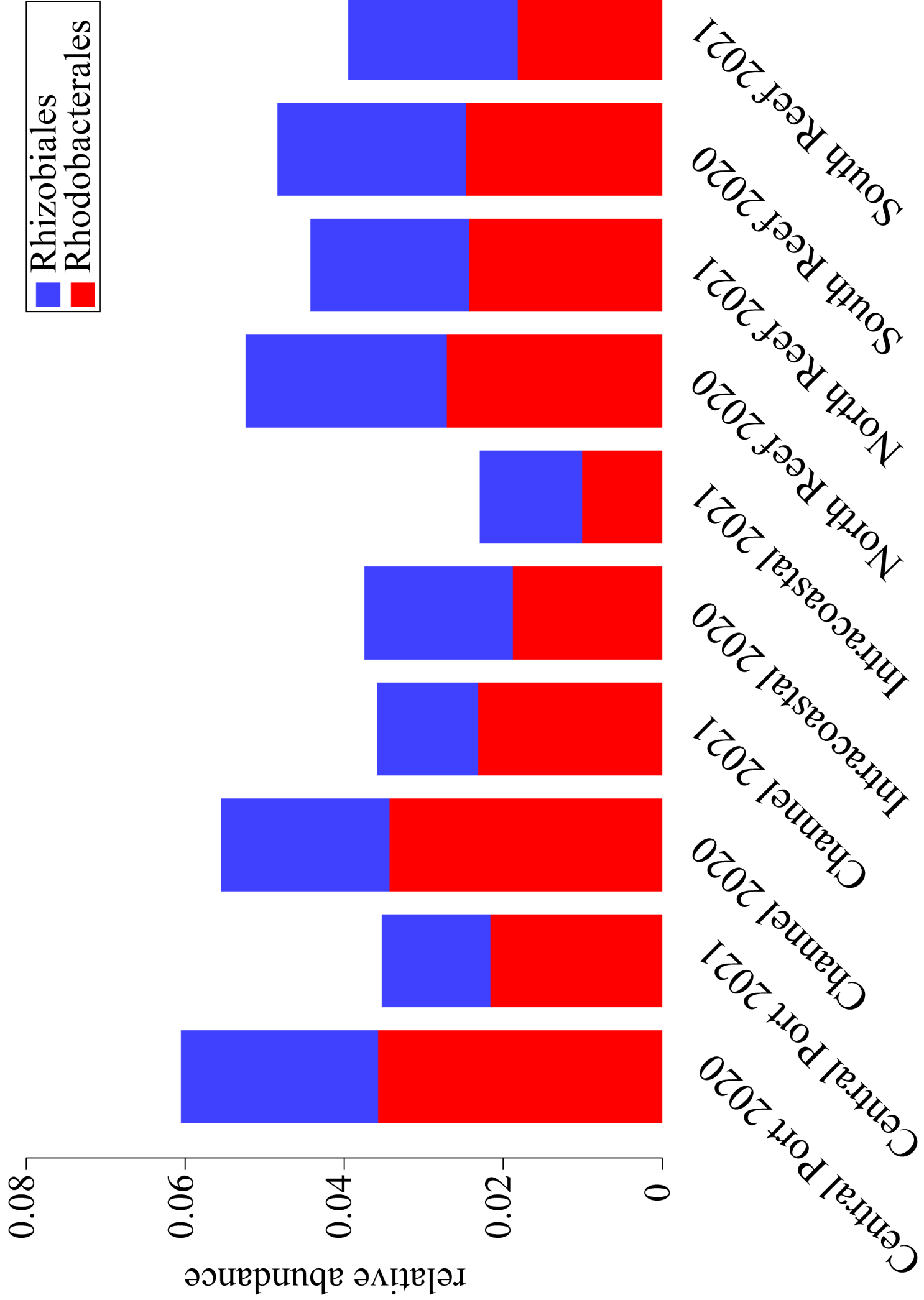

Supplement: Supplemental Information 6 [file peerj-11-14288-s006.pdf]

A.

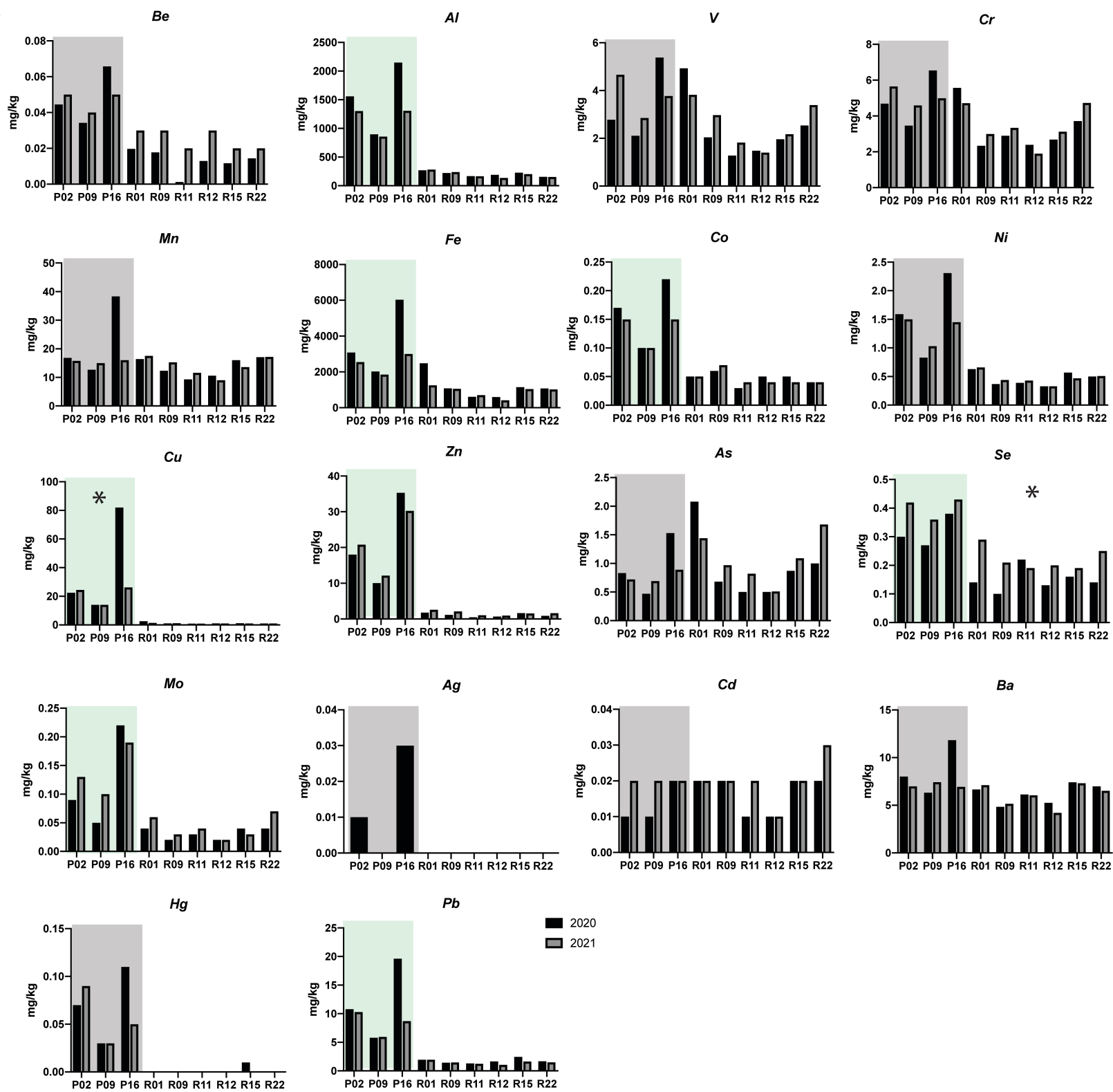

B.

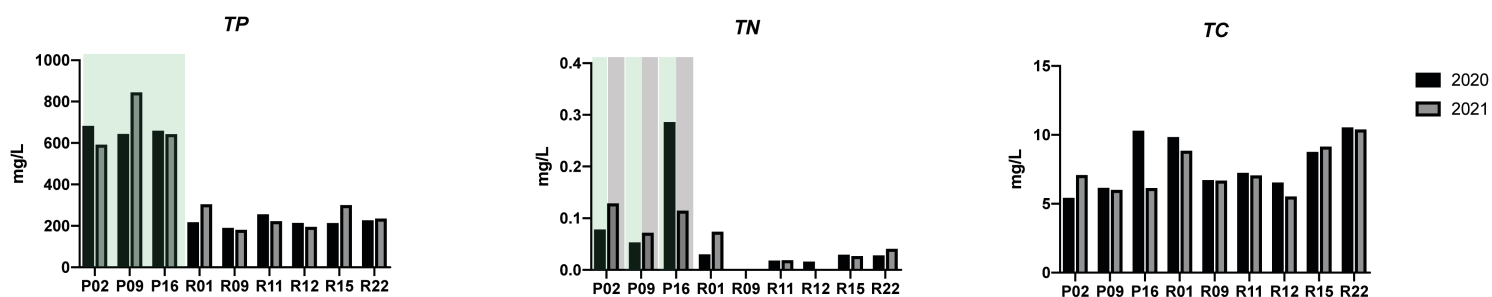

Supplement: Supplemental Information 7 — The shading highlights the PEI samples taken for each element. If the shading is green, it indicates there were statistical differences in the concentration of that element in the PEI between 2020 and 2021, and the gray shading indicates there were no differences between 2020 and 2021 for that element in the PEI. An asterisk indicates if there were statistical differences in that element in the reef in after dredging in 2021. Statistically significant differences between the PEI and reef, or between years at either site were determined using one-way ANOVA with a p value cut-off of 0.05. [file peerj-11-14288-s007.pdf]
